# Innovative strategies for annotating the “relationSNP” between variants and molecular phenotypes

**DOI:** 10.1186/s13040-019-0197-9

**Published:** 2019-05-14

**Authors:** Jason E. Miller, Yogasudha Veturi, Marylyn D. Ritchie

**Affiliations:** Department of Genetics, Institute for Biomedical Informatics, Perelman School of Medicine, University of Pennsylvania, 3400 Civic Center Blvd., Philadelphia, PA 19104 USA

**Keywords:** Precision medicine, Variant, Mutation, SNP, Synonymous, Non-coding, Non-synonymous, Machine-learning, Deep-learning, Resource, Software, Tools

## Abstract

Characterizing how variation at the level of individual nucleotides contributes to traits and diseases has been an area of growing interest since the completion of sequencing the first human genome. Our understanding of how a single nucleotide polymorphism (SNP) leads to a pathogenic phenotype on a genome-wide scale is a fruitful endeavor for anyone interested in developing diagnostic tests, therapeutics, or simply wanting to understand the etiology of a disease or trait. To this end, many datasets and algorithms have been developed as resources/tools to annotate SNPs. One of the most common practices is to annotate coding SNPs that affect the protein sequence. Synonymous variants are often grouped as one type of variant, however there are in fact many tools available to dissect their effects on gene expression. More recently, large consortiums like ENCODE and GTEx have made it possible to annotate non-coding regions. Although annotating variants is a common technique among human geneticists, the constant advances in tools and biology surrounding SNPs requires an updated summary of what is known and the trajectory of the field. This review will discuss the history behind SNP annotation, commonly used tools, and newer strategies for SNP annotation. Additionally, we will comment on the caveats that distinguish approaches from one another, along with gaps in the current state of knowledge, and potential future directions. We do not intend for this to be a comprehensive review for any specific area of SNP annotation, but rather it will be an excellent resource for those unfamiliar with computational tools used to functionally characterize SNPs. In summary, this review will help illustrate how each SNP annotation method impacts the way in which the genetic and molecular etiology of a disease is explored *in-silico*.

## Introduction

Scientific endeavors in human genetics, molecular biology, biochemistry, and bioinformatics have been progressively converging in order to more precisely describe how DNA variation explains differences in traits and diseases. Single base changes called single nucleotide polymorphisms or SNPs, along with changes where DNA has been inserted (e.g. insertions) or deleted (e.g. deletions), which are referred to as indels have been popular forms of genetic variation to investigate. Another form of variation is in terms of copy number variants (CNVs), where large portions of the genome are duplicated or deleted. These variants in our DNA differentiate us in terms of how we define our ancestry, the unique traits we all have, what diseases we inherit or sporadically develop, along with what medicinal therapies may be best suited for us. Therefore, being able to characterize how this portion of the genome influences life is of significant value to those trying to not only understand biology, but also developing tools and methods for improving health.

Most of what we know about genetic variation on a genome-wide scale (as opposed to studying individual genes) has accumulated over the past 20 years, starting with the human genome project. As the name suggests, the human genome was sequenced as a part of the human genome project [1, 2]. While it was funded by the National Institutes of Health (NIH) and Department of Energy (DOE), it was also informally a product of international collaborations [3]. Some of the regions that were highly palindromic and repetitive were not able to be accurately sequenced and are still being investigated today, such as the HLA region [4, 5]. Additionally, segmental duplications are a type of structural alternation of the genome that can share high sequence similarity with one another and require special attention when investigating [6]. Key findings included the number of genes, base pairs, and higher levels of evolutionary conservation than previously thought. The human genome project only represented a small number of individuals, but scientists knew that it was variation in the genome that would lead us to an improved understanding of how genetic diversity contributes to disease.

Since we have two copies of each chromosome, we can be heterozygous or homozygous at any location in the genome, but these differences have significant implications with respect to the resulting phenotype. To identify alterations in haplotypes the HapMap project was started [7, 8]. Since individuals of the same ancestry share more haplotypes in common than those of different ancestry, the HapMap project genotyped individuals from the United States, Yoruba, China, and Japan in Phase I. Phase II/III expanded to genotyping individuals from Nigeria, two regions in Kenya, Italy, along with African-Americans, Chinese-Americans, and Mexican-Americans for a total of 11 populations [7]. Characterizing ancestral groups has been valuable to those interested in human migration and/or disease and continues to be a rich source of genetic information. The data was deposited into the publicly available website, dbSNP [9]. Each SNP was provided a reference SNP cluster ID number or rsID for short. Other consortia such as the National Institute of Environmental Health Sciences (NIEHS) Environmental Genome Project has explored how people’s susceptibility and outcomes related to environmental exposure may be influenced by SNPs [10]. Moreover, the 1000 Genomes Project, published its first pilot study in 2010 and eventually was able to ultimately sequence over 2000 genomes [11]. Whole-genome sequencing provided a much more granular characterization and provided data to identify more insertions, deletions, and rearrangements in the genome. It provides > 99% of variants that have a frequency of greater than 1% in the population [11]. While some of these projects pointed towards 0.1% of our 3 billion base pairs varying between humans [11–13], a recent study that sequenced 910 individuals of African descent found 300 million base pairs missing from the reference genome [14]. These results suggest multiple reference genomes are likely necessary and scientific research would benefit by creating more diverse cohorts. Fortunately, the Exome Aggregation Consortium (ExAC) released 60,706 exomes from a diverse population [15]. The same consortium later released 125,748 exomes and 15,708 whole genomes in total as a part of the Genome Aggregation Database (gnomAD) which also came from diverse ancestral backgrounds [16]. A more thorough description of the technological advances made by these consortia and others has recently been described [17].

Researchers assumed that the human genome project, HapMap, 1000 Genomes project and other similar studies, would resolve many questions with respect to connecting a SNP to a trait or disease, however this was not the case. Rather, by sequencing the genome, scientists now had what is analogous to a book that contains the history of humans; they understood the alphabet, but weren’t sure how to translate the words into meaning. Even finding the “words”, which one might think of as genes is still an ongoing effort [18]. The biggest issue was trying to connect these SNPs to diseases. However, there were many hints based on previous molecular and biochemical studies that preceded these genome-wide analyses. Enough was known about mRNA expression to at least infer that connecting gene expression to SNPs or expression quantitative loci (eQTL) could help with finding a relationship between SNP and functional effect. Moreover, variation in the coding region could cause highly predictable changes such as amino acid changes or pre-termination stop codons (e.g. non-sense mutations).

In this review, we will describe some of the most commonly used methods along with new methods that innovatively annotate SNPs. Annotation methods can broadly be characterized by whether or not they annotate variants based on location (i.e. non-synonymous or 5′ untranslated region) or if they are more quantitative and intend to make inferences about pathogenicity or deleteriousness of the variant [19]. We will compare and contrast these methods with respect to different regions of the genome, but primarily focus on qualitative annotations. Variant annotation databases, which collate disease relationships and other characteristics of variants, such as OMIM and ClinVar, have previously been reviewed [20] and are not included in this review. Both coding and non-coding regions will be topics explored herein. While synonymous variants are often left out the analysis, we will describe their biological significance, along with how older and newer methods have annotated these features with varying degrees of success. We will also offer advice in terms of how these methods could be applied to genetic studies. Finally, we will discuss future directions of where SNP annotations could go next. This review will primarily cover annotations for SNPs, while reviews of annotations and identification of indels and CNVs can be found elsewhere [21].

## Annotating coding regions seems straightforward, but caveats are abundant

### The importance of annotating SNPs that influence protein sequence

Our understanding of how a protein coding gene is transcribed and translated into a protein along with how it performs its function has significantly influenced how SNPs are annotated. In parallel with those interested in identifying new SNPs, there have been many focused on the fundamental mechanisms by which gene expression is regulated and the eventual effect on the trait or disease. The cross pollination between these fields of study has led to an appreciation for characterizing how SNPs influence gene expression, and thus an interest in utilizing what is known on a molecular level to annotate SNPs [22]. Moreover, annotations of non-synonymous changes have always been a primary focus of some of the earliest methods.

Annotating SNPs that cause a change in the amino acid sequence (e.g. non-synonymous variants) is critical to helping us understand the relationship that SNPs have with traits and diseases. Non-synonymous variants can be in the form of single amino acid changes, frameshifts (i.e. multiple base pairs added or subtracted from the gene), stop loss, and stop gained (e.g. non-sense) variants. These types of changes can impact enzymatic activity, protein interactions with other molecules, and interactions within the same protein molecule. Additionally, alternative splicing can be affected by these changes as well through non-synonymous changes to splice sites, thus impacting the amino acid and entire exons as well. Most genes have alternative splicing isoforms and this has also led to a subset of annotations that incorporate whether the SNP position is located at a splicing junction [23]. Variants affecting these regions can thus impact inclusion and exclusion of introns and exons from being translated. Thus, many of the annotations and tools used to annotate SNPs have to be flexible in terms of what they can annotate.

### Popular methods for annotating coding variants

For over a decade tools have been developed to annotate variants. In that time, a handful have become the most popular. Additionally, they utilize a combination of evolutionary conservation, published resources, expert knowledge, and computational predictions to characterize the importance of the variant on a molecular level, as well as its ability to contribute to a pathogenic phenotype. While some of them require amino acid information as the input, they are still commonly used to annotate non-synonymous variants. Sorting Intolerant From Tolerant (SIFT) uses evolutionary conservation to score whether or not a non-synonymous change could potentially affect the function of a protein and thus impact a phenotype [24]. SIFT performs a PSI-BLAST search to collect information about the likelihood that the amino acid substitution has been observed and is thus tolerated in an evolutionary sense. One of the weaknesses of this tool is that it does not incorporate the protein structure into the prediction.

PolyPhen is another annotation tool which primarily uses sequence conservation, however it includes information pertaining to 3-D structure and contacts with other residues as well [25]. While PolyPhen performed well against other methods in a benchmark study, the accuracy tended to vary based upon the structural class of the protein [26]. SnpEff is a tool that can annotate both non-coding and coding regions and includes output that describes the effect (i.e. High, Moderate, Low, or Modifier) [27]. Some advantages of using SnpEff include its speed, ability to be integrated with other open-source programs like Galaxy, and its ability to accommodate genomes aside from human [27]. SnpEff does have some weaknesses including the lack of miRNA structural annotations, transcription factor binding site scoring, and splicing annotations, but these may not be a significant disadvantage depending on the application. Tools like dbNSFP were able to combine many of the programs mentioned and others (i.e. SIFT [24], Polyphen2 [25], LRT [28], PhyloP [29], and MutationTaster [30]) into a single database for annotation and filtering purposes [31]. Recently, dbNSFP has expanded to include more databases and tools (i.e. FATHMM [32], MutationAssessor [33], and others), and provides the ability to annotate splice-site variants as well [34]. Variant Effect Predictor (VEP) was developed by Ensembl to annotate both coding and non-coding variants (Table 1), and in addition to SNPs can also annotate indels and CNVs larger than 50 bp [35]. It has the advantage of allowing for plug-ins from other resources such SIFT and PolyPhen annotations. One way to help support that an allele is pathogenic is by checking if it is selected against, or in other words, it has low frequency in the population. Tools like VEP and dbNSFP, among others, have the ability to easily search through population based data like the 1000 Genomes Project and gnomAD [16], and others to filter for these rare SNPs.

**Table 1:** Methods to characterize SNPs across coding and non-coding regions

Methodology	Tool	Website
Annotates and/or filters SNPs, insertions, and deletions for any study design.	ANNOVAR [52]	http://annovar.openbioinformatics.org/en/latest/
	Biofilter [165]	https://ritchielab.org/software/biofilter-download-1
	Myvariant.info [53]	http://myvariant.info
	^a^SnpEff [27]	http://snpeff.sourceforge.net
	SNPnexus [166]	http://www.snp-nexus.org
	VCFanno [167]	https://github.com/brentp/vcfanno
	VEP [35]	https://useast.ensembl.org/info/docs/tools/vep/index.html
Annotates GWAS SNPs based on functional information and visualizes results	FUMA [137]	http://fuma.ctglab.nl
	INFERNO [136]	http://inferno.lisanwanglab.org/index.php
Associate SNP with phenotype information mediated by gene expression data.	COLOC	http://cran.r- project.org/web/packages/coloc
	eCAVIAR [168]	http://genetics.cs.ucla.edu/caviar/index.html
	enoloc [122]	https://github.com/xqwen/integrative
	FOCUS [134]	https://github.com/bogdanlab/focus
	PrediXcan [128]	https://github.com/hakyimlab/PrediXcan
	SMR [169]	https://cnsgenomics.com/software/smr/#Overview
	TWAS [125]	http://gusevlab.org/projects/fusion/
	UTMOST [132]	https://github.com/Joker-Jerome/UTMOST/
Methods that generated predictive scores that suggest a SNP may influence a molecular phenotype.	CADD [40]	https://cadd.gs.washington.edu
	DANN [170]	https://cbcl.ics.uci.edu/public_data/DANN/
	FIRE [171]	https://sites.google.com/site/fireregulatoryvariation/
	LINSIGHT [142]	https://github.com/CshlSiepelLab/LINSIGHTCA

^a^SNPeff also has the ability to perform visualization

The use of machine learning (ML) has been a growing area of interest in the genetics community, and more specifically for those interested in characterizing SNP function and potential pathogenicity [36–39]. ML is a broad term used for algorithms that learn from a training dataset to improve the mathematical model prediction accuracy on a test data set. Often, these methods use sequence conservation, amino acid physiochemical properties, gene regulatory annotations, allele frequency among sub-populations, and even the output of other tools. Combined Annotation-Dependent Depletion (CADD) annotations were derived from a support vector machine (SVM), a commonly used ML algorithm, to generate scores for 8.6 billion possible single nucleotide variants (SNVs) in the human reference genome based on 63 annotations that described conservation, gene regulatory information, and population frequencies [40]. Methods like PhD-SNP [41] and SNPs&GO [42] also use an SVM to generate scores for predicting potentially deleterious effects of variants. In addition to SVM, random forest, feed forward neural networks, and naive Bayes are commonly used ML methods when training models to predict deleterious variants. MutPred [43] and MutPred2 [44] are examples that use random forest, while SNAP [45] and SNAP2 [46] use neural networks, and MutationTaster [47] uses naive Bayes. On the other hand, VEST can generate *p*-values for each variant in addition to a score so that they can be aggregated for gene-level information [48]. Many of these tools and others have been reviewed and compared to one another [20].

Another category for SNP annotation strategies encompasses “meta-predictors”, which utilize multiple methods to generate a prediction of potential pathogenicity. REVEL (rare exome variant ensemble learner) was able to better characterize rare missense variants as neutral or potentially pathogenic compared to CADD by using random forest [49]. While it is unclear how using random forest as opposed to other methods may change the results, it is likely that designing the training and testing sets to have no overlap and include features that are most relevant to characterizing rare missense variants greatly helped in characterizing pathogenicity. Other popular methods that fall into the meta-predictor category include MetaLR [50], MetaSVM [50], and Condel [51]. These meta-predictions should be thought about in contrast to tools that aggregate data from multiple sources so that you can access multiple databases using single tool. Some of the tools that aggregate information from other tools without generating a new predictive score include ANNOVAR [52], dbNSFP [34], VEP [35], and Myvariant.info [53] (Table 1).

Another facet of annotating variants in coding regions is with respect to three-dimensional space by studying how SNPs impact protein-protein interactions. A recent review covering the prediction of functional impact on non-synonymous variants recently addressed structural annotations from a historical perspective [54], but we will touch on a few points here related to newer methods related to protein-protein interactions. The POINT (protein structure guided local test) method uses a kernel machine framework to generate variant context in 3D space, along with information pertaining to the impact variants have on stability and protein-protein interactions [55]. Another tool called BindProf was developed which uses machine-learning to incorporate information about sequence similarity, protein-protein interfaces, along with evolutionary, and structural information to predict how mutations can impact protein-protein interactions [56]. Visualization of protein-protein interactions can be performed to assist with interpretation and provide additional analyses. There are standalone methods like BioLayout3D [57], Arena3D [58], and NAViGaTOR [59] or you can use a plugin for cystoscope called 3DScapeCS [60]. The method OmicsNet is a web interface that contains many of the features of previous tools, along with allowing for more complex interactions, enrichment analysis, and module detection [61]. However, annotating variants with respect to 3D space remains an open challenge for the bioinformatics and protein structure fields [62]. These methods assume that structural or protein-protein information exists for your SNP of interest or similar regions that are close enough to make a useful prediction. So, if a SNP falls into a region that cannot be crystalized or there is limited structural information, the results may be difficult to interpret. Having said that, as crystal structures start to include larger proteins at higher resolution, along with improvements in other structural data such as cryo-EM and NMR, we will likely see improvement in these tools as they evolve to fully take advantage the rich information available.

### Challenges for annotating the coding region

Although these annotation tools have been successfully used in a variety of applications, there are not only discrepancies between methods, but unresolved questions that no current method has properly addressed as it relates to non-synonymous variants. For instance, there are multiple annotations that can be used for each gene, depending on the method and splicing isoform, however it is unclear which annotation is best [63, 64]. While these popular methods can annotate all types of coding variants, there are a number of more specialized annotation tools that focus more on splicing [65]. These splicing methods utilize a number of features to predict splicing including thermodynamics (Analyzer Splice Tool [66, 67]), detection of binding motifs (SFmap [68]), and sequence motifs associated with splicing (FAS-ESS [69]) (and reviewed in [65]). These new tools and annotations will be important for improving the accuracy and interpretation of annotations within coding regions [70]. Moreover, each of these methods predicts different SNPs as potentially pathogenic so there may not be a one size fits all solution when designing a study. For this reason, it is beneficial to test multiple annotation strategies within a single study, thus, the results will not be biased by a single method, and results that replicate may be more robust [71].

## Capturing the differences among synonymous variants

### Why are synonymous variants often ignored in human genetic studies?

The way in which the genome codes for proteins is considered degenerate since there are 64 codons that only code for 20 amino acids. Codons that are translated into the same amino acid are referred to as synonymous. Thus, a synonymous variant or mutation changes the codon but not the amino acid. Often, synonymous variants and mutations are either assumed to be neutral, all have the same effect on the protein, or left out of the analysis entirely [72]. Though it is an inconvenient truth for those willing to throw out the synonymous variants, geneticists and biochemists have demonstrated for over 30 years that synonymous codons are not used at equal ratios and that this phenomenon represents what is called “codon bias” and that this feature of biology has implications in gene regulation and explaining the etiology of disease [73]. Moreover, synonymous variants have similar effect sizes as non-synonymous variants associated with disease, thus greater attention should be spent investigating the mechanism behind these effects [74].

### Synonymous codons can be annotated with respect to codon bias in terms of frequency and optimality

Synonymous variants can be characterized by their impact on codon bias, mRNA processing, translation, or protein structure. The earliest measure of codon bias was defined by the relative synonymous codon usage (RSCU) index. It is calculated using the equation RSCU = SNc/Na, where S is the number of synonymous codons for an amino acid, Nc is the frequency of the codon within the genome and Na is frequency which the amino acid that Nc in the genome [65, 75]. Calculating the change in RSCU (e.g. ΔRSCU) is the most informative as it tells whether or not a mutation or SNP alters the RSCU and possibly translation. SNPs that affect RSCU are associated with a number of diseases including Pulmonary sarcoidosis, Hemophilia B, non-small-cell lung carcinoma, cervical and vulvar cancer, and both adult and child attention deficit/hyperactivity disorder (ADHD) [75]. Another mechanism that explains why synonymous codons differ is codon optimality, which describes interactions with cognate tRNAs. Codon-tRNA interactions that are stronger are considered optimal and weaker interactions are non-optimal, both of which affect translation rates. It has previously been illustrated that annotating codons in terms of optimality has provided new insights into cancer and Alzheimer’s disease [76, 77].

### Splicing, mRNA folding, and stability annotations can be employed to investigate synonymous variants

In addition to codon bias that affects translation, annotating SNPs that affect splicing, miRNA binding sites, and mRNA structure have also proved useful when dissecting synonymous variants. Splicing is one of the most common mechanisms by which annotations have been designed to characterize synonymous codons. These tools are generally interested in the effect on either 5′ or 3′ splice sites or splicing regulatory elements. Many of the tools used to investigate the effects of splicing on synonymous codons have been reviewed, however a number of new methods have recently been published. UMD-predictor is able to annotate cDNA substitutions by incorporating protein domain information, conservation across species, allele frequency in the human populations, and potential impact on splicing [78]. Although UMD-predictor has been suggested to on its own to have more accurate predictions than other commonly used methods, a recent report suggested UMD along with SIFT and PolyPhen can be used to characterize mutations in the gene *GNB5* which can cause autosomal-recessive multisystem syndrome with sinus bradycardia and cognitive disability [79]. On the other hand, a newer method called TraP (Transcript-inferred Pathogenicity) can detect pathogenicity of synonymous variants that affect splicing by measuring their impact on splice sites and intronic regions that splicing factors bind [80]. Moreover, TraP was validated using semi-quantitative RT-PCR, suggesting the results can move beyond providing only in silico results.

Codon bias can also impact mRNA secondary structure, which in turn can alter mRNA stability and protein binding, thus impacting gene expression. The mechanisms by which mRNA secondary structure can influence gene expression is mediated through a variety of mechanisms including transcription, splicing, stability, translation, miRNA-mRNA interactions, mRNA localization, and protein expression [81]. Genetic variation affecting mRNA secondary structure has been linked to Schizophrenia, and may have a physiological impact on a person’s kidneys, skin, lungs, heart, immune system, and neurological traits [75, 82]. To annotate changes in the secondary structure of mRNA, several in silico methods have been developed, including mFold [83], Kinefold [84], remuRNA [85, 86], and RNAfold [87]. mFold, Kinefold, and RNAfold can simulate a static mRNA secondary structure based on free energy kinetics and simulations. Whereas remuRNA is actually designed to determine how a rare SNP will impact the mRNA secondary structure relative to a population [86].

#### Methods and challenges of investigating the impact synonymous variants have on translation and protein structure

A predominant theme among those investigating the effects synonymous variants have on genes is by measuring or predicting the changes specifically to translation, protein structure, and folding. The rate of translation is important for protein fidelity and folding. There are multiple mechanisms and processes that affect translation including mRNA structure, mRNA stability, and sequence motifs, many of which have been proposed to be effected by codon bias [65]. While synonymous genetic variation in humans is often lumped together as a single type of variant, a number of currently available tools and databases make it accessible to categorize synonymous variants by potential effects on gene expression with respect to protein translation and structure. One tool that can prioritize synonymous variants based on mRNA structure and protein function is regSNP [88]. This method evaluates the potential effect a SNP has using a machine-learning based approach. Although the number of tools that can annotate synonymous variants beyond whether or not they are synonymous is sparse, there are a number of databases and publications which provide annotations to manually annotate the variants. Recent work that annotated SNPs based on spatial distribution used CATH (Class Architecture Topology Homology) to divide synonymous variants into which domain they fell in within a given protein [89–91]. While no statistically significant differences were seen between synonymous variants among the varying protein domains it offers some insights into how synonymous variants can be characterized further.

## Exploring non-coding regions using data from consortiums

### Consortiums have generated most data used for annotating non-coding regions

The human genome project provoked many questions regarding the function of the non-coding regions of the genome and how gene expression was being regulated. One of the most important discoveries was that most of the genome did not code for proteins. Furthermore, genome-wide association studies (GWAS) have found that most disease associated SNPs are not within genes, suggesting the non-coding regions have medical relevance [92]. However, after evidence was building that these non-coding regions were important, there were only a handful of studies that had actually investigated how they were regulated on a genome-wide scale, let alone how they might be impacted by genetic variation. To address these and other questions, multiple consortia were created to collect biological samples from human and model organisms to perform a variety genome-wide functional experiments.

The Encyclopedia of DNA elements or ENCODE, was first launched in 2003 as a large group of scientists from multiple institutions that would generate new experimental and computational methods to characterize and annotate beyond the coding regions of the genome [93]. Within a decade, ENCODE had developed many new methods that were able to annotate transcription factor (TF) binding sites, chromatin states, RNA-protein interactions, DNA methylation, gene expression, RNA-protein interactions, and three-dimensional chromatin interactions [94]. Additionally, a number of off-shoot programs began because of the success of ENCODE, such as NIH ROADMAP, which characterized chromatin marks throughout the genome, and modENCODE, which performed similar techniques as ENCODE but in model systems such as yeast, worms, and flies [95–97]. This information has been critical to annotating SNPs with functional information.

Although ENCODE helped resolve the biochemical mechanisms by which genes are regulated on a genome-wide scale, it remained unclear how genetic variability affected gene expression. In other words, how do SNPs in non-coding regions impact RNA levels? And if a SNP does impact gene expression, what is the tissue context (i.e. brain)? To address this question, and others, the Gene-Tissue Expression consortia (GTEx) was started in 2013 to sample tissues from hundreds of individuals across many tissue types [98]. Some of the most valuable pieces of information that came from GTEx were in the form of expression quantitative trait loci (eQTL) and eGenes. eQTLs are SNPs that explain variability in gene expression across individuals, and eGenes are the genes whose expression is impacted by said eQTLs. While this review will not delve deeply into consortia that are more disease or trait specific, we would be remiss to not at least mention them. For instance, The Cancer Genome Atlas (TCGA) and International Cancer Genome Consortium (ICGC), established in 2006 and 2007, respectively, have generated many annotations and datasets for the purpose of investigating mutations leading to cancer, while the Genetic Investigation of ANthropocentric Traits (GIANT) and Global Lipids Genetics Consortium (GLGC) have identified novel SNPs associated with anthropomorphic and lipid traits, respectively [99–101].

### Methods to annotate non-coding regions using information gleaned from chromatin architecture

Even though ENCODE did not investigate genetic variation, the transcription factor and chromatin annotations have been invaluable to those interested in annotating SNPs in non-coding regions. One of the reasons ENCODE was successful was that researchers hypothesized that regions of the genome that do not code for genes, but regulate the expression of genes, are likely to harbor disease causing mutations that affect protein-DNA interactions. Experimental methods such as ChIP-seq, DNase-seq, and bioinformatics methods that searched for protein binding motifs to detect these interactions throughout the genome were employed in ENCODE [93]. The hypothesis that these regions are important, while at one point controversial, is now supported by mounting evidence that illustrates most non-coding SNPs impact gene expression [102]. One mechanism by which ENCODE tested, was the ability of a SNP to impact transcription factor binding. Two popular databases used to annotate non-coding variants are RegulomeDB and HaploReg [103, 104].

HaploReg is a tool that annotates variants with respect to ENCODE data either through its online interface, or by downloading the annotations that the authors collected [104]. HaploReg is a fast and convenient method for finding if the SNP of interest or nearby loci are located in regions defined as promoters, enhancers, or protein binding sites. It can also tell if the region is in a DNase sensitive region or is located near GWAS SNPs from NHGRI/EBI GWAS catalog (https://www.ebi.ac.uk/gwas/). Furthermore, it performs a statistical test that can provide insights as to what cell type the SNPs are likely to be most relevant. HaploReg has successfully characterized SNPs associated with cardiovascular disease, autoimmune disorders, cancer, diabetes, and neurological disorders [105, 106]. RegulomeDB, which was published around the same time as HaploReg, can annotate SNPs with scores based on the amount of overlapping annotations from ENCODE [103]. For instance, if a SNP overlaps with an eQTL, TF binding site, a DNA motif, DNase footprint, a DNase peak, it is given a score that suggests it is likely to affect binding linked to the expression of a gene. Whereas if a SNP only overlaps with one or two forms of evidence such as a motif hit or TF binding site it is scored as unlikely to affect binding. RegulomeDB has characterized SNPs associated with cancer [107, 108], Alzheimer’s disease [109], and Schizophrenia [110], just to name a few. Both HaploReg and RegulomeDB are very similar, however the biggest difference is that HaploReg has a built-in function that suggests tissue type using a statistical method whereas RegulomeDB does not. On the other hand, RegulomeDB has a scoring method for providing the user with information about the likelihood of the SNP having an impact on gene expression. Finally, while RegulomeDB only annotates SNPs that you provide it, HaploReg will also annotate variants that are in linkage with the SNPs provided for the input. Both methods are useful, and these features can be an advantage or disadvantage depending on what questions are being addressed, so it is important to define a question then pick the tool. Both methods have been cited over 700 times and are still used regularly in papers (pubmed.gov).

Another category of methods that can be used characterize non-coding variation is through enrichment analysis. These methods are often attempting to investigate the role of distant regions of the genome coming together in 3D space. These methods investigate chromatin architecture either through the use of ChIP-seq data on proteins that regulate chromatin looping or chromatin capture results like those from Hi-C [111]. The tool GREAT can take a set of input genomic regions and a gene ontology annotation then test for a significant enrichment using a binomial test [112]. While it defines these regulatory regions as up and downstream of the gene, it can incorporate experimental data as well. The method TAD_Pathways integrates GWAS data and published topologically associated domain (TAD) boundaries from human embryonic stem cells to perform pathway analysis [113]. Interestingly, the authors experimentally validated the gene *ACP2* as playing a novel role in human osteoblast development, even though it was not the nearest gene nor in the same LD block as the GWAS SNP [113]. Most recently, the tool Biological Enrichment of Hidden Sequence Targets (BEHST), was developed to perform gene-set enrichment analysis using 3D chromatin architecture information and genomic regions as input [114]. BEHEST has greater precision and accuracy relative to existing methods when detecting enriched gene ontology terms for a given set of enhancers [114]. While its first application was using enhancers, the authors suggest the software can be used to investigate long range chromatin interactions that connect SNPs to pathway level information.

A weakness of ENCODE, NIH Roadmap, and some of the Hi-C data discussed above was that most of the samples came from cell lines of the same individuals, which meant it would be very difficult to understand how SNPs had an effect on gene expression from those samples alone. To overcome this obstacle, the gene-tissue expression or GTEx consortium was established in 2010 to investigate how gene expression varies across individuals without disease [92]. GTEx enabled researchers to investigate genetic variation in the context of expression. SNPs that affect expression of a gene are known as expression quantitative trait loci (eQTL) and are now being used to annotate SNPs in the context of gene expression. Additionally, ENCODE did not consider the potential affects that ancestry of the donor could have on their experiments. Thus, future studies should investigate the role of ancestry on a consortia level.

### Utilizing eQTL data for interpretation of genome-wide association studies

Unlike many of the other methods discussed in this review that focused on individual SNPs in a trait-agnostic manner, the methods in the following section require GWAS data (summary-level or individual-level). While GWAS has been fruitful in finding associations between common variants and phenotypes, these SNPs often fall well outside of coding regions making it difficult to identify which genes these SNPs regulate [115]. Therefore, the primary goal of GTEx was to create a data resource to study the relationship between genetic variation and gene expression across multiple tissues [116]. Additionally, it wanted to disseminate tissue samples, data, new methods, and scientific knowledge [116]. In 2015 and 2017, they released a number of high-profile papers which resolved fundamental questions related to genetic variation and tissue specificity. Additionally, the GTEx consortium and other groups which used GTEx data have developed a number of tools and strategies to annotate variants using this new source of data.

The primary annotation that GTEx provides are eQTLs. A recent release from GTEx identified 24,886 *cis*-eQTLs and 673 *trans*-eQTLs, but more importantly it alluded to the difficulties in detecting SNPs that are most influential with respect to expression of a specific gene [102]. For instance, 90% of common GWAS SNPs are eQTLs for one or more genes (some are associated with 30 or more nearby genes) while only 40% of GWAS signals co-localize with the nearest gene [102]. Since it is a logistical challenge to generate gene expression data for every GWAS, there is a growing area of research focused on developing methods that utilize GTEx in other contexts, specifically those that are interpreting GWAS results. Tissue specificity, LD structure, and the overall abundance of eQTLs pose challenges when developing these methods. Most methods that integrate eQTL and GWAS information are either in the form of colocalization or transcriptome-wide association analysis. Thus, the main advantage of these methods is that gene expression and genotype/phenotype information can come from completely independent individuals.

Colocalization methods test for the co-occurrence of signals in both eQTL and GWAS datasets. Two of the earliest examples of that investigated colocalization utilized lymphoblastoid cells lines (LCL) from the HapMap project [117, 118]. The regulatory trait concordance (RTC) method had the advantage of accounting for linkage disequilibrium (LD), and being able to test for both cis and trans effects [117]. Interestingly, immunity-related traits were over-represented, suggesting the source of RNA expression data (e.g. LCL) contributed to which traits were associated. Another study that investigated the co-occurrence of GWAS and eQTL signals in LCLs concluded that trait-associated SNPs are more likely to be eQTLs [118]. Later on, COLOC was developed, which uses a novel Bayesian statistical test to evaluate the co-occurrence of two association signals that share a causal variant. This method can identify novel shared mechanisms by focusing on a single genomic region at a time while accounting for LD at that locus. It also has the advantage of using summary-level data (SNP *p*-values and their minor allele frequencies or SNP effect estimates and standard errors) [119]. A conceptually similar approach called Sherlock [120] was developed around the same time. More recently, eQTL and GWAS Causal Variant Identification in Associated Regions or eCAVIAR was developed which also uses summary statistics like COLOC but makes the added assumption of having multiple causal SNPs in a locus unlike COLOC which assumes a single causal variant [121]. A more flexible approach called enrichment estimation aided colocalization analysis (enloc) [122] can perform enrichment in addition to fine-mapping and colocalization. All these methods output posterior probabilities that can help infer whether a shared variant is “plausible”; it is to be noted that they cannot help to detect the actual causal variant. Colocalization methods have also been employed to perform systematic genome-wide scans on large phenotyped datasets to detect genetic variants that influence pairs of traits, instead of gene expression and trait [123].

Transcriptome-wide association methods evaluate the association between a SNP and a trait through measured or predicted expression [124]. These methods are sometimes collectively called TWAS, which we will apply here, but it should be noted there is also a tool called “TWAS” [125]. TWAS is conceptually similar to colocalization methods. In their analyses Gusev et al. detected slightly higher power with TWAS than COLOC since it can better capture LD even among “untyped” variants, something that colocalization methods are not designed to achieve. On the other hand, TWAS cannot provide a basis for whether the “causal” variants in GWAS and eQTL datasets colocalize. TWAS can calculate weights for SNPs based on gene expression data (e.g. GTEx data), then apply those weights to GWAS summary statistics to identify expression-trait associations. TWAS reduces the multiple test burden and increases power relative to the standard GWAS by (a) testing for associations of a trait with genes rather than millions of SNPs, whose effect is possibly mediated through changes in gene expression and (b) by imputing *cis*- gene expression from a small set of genotyped individuals into a much larger set of phenotyped individuals using their SNP information. TWAS has been effective at identifying new genes associated with complex traits that were unlikely to be detected through their proximity to the causal SNPs [126]. However, there are a few caveats to using these TWAS approaches. If the effect a SNP has on a trait is mostly mediated through a mechanism that does not change transcript levels it will not be detected. Secondly, based on simulations, TWAS has less power when the effect is pleiotropic in nature [127]; approaches such as Summary-based Mendelian Randomization (SMR) and a post-filtering step called Heterogeneity in Independent Instruments (HEIDI) proposed by Zhu et al. are designed to detect pleiotropic effects. Additionally, those using TWAS often use it to predict expression when no gene expression data is available, especially using the PrediXcan method [124, 128]. While PrediXcan can predict some genes accurately, it does not predict them all well even after including more putative eQTLs or integrating multiple datasets [129]. It therefore may be best to use TWAS to prioritize genes and eQTLs as opposed to attempt to replace actual data until more robust imputation strategies are developed. Other methods have also attempted to extend TWAS to further estimate a genetic correlation between trait and gene expression (using GWAS and eQTL data) while using bidirectional regression to investigate any potential causal direction for genetically correlated trait pairs [130].

Recently, the authors of PrediXcan developed S-PrediXcan, which is a version of PrediXcan that can use summary-level information [131]. They developed a generalized framework called MetaXcan that can incorporate the results of multiple TWAS and colocalization methods to investigate the gene to phenotype relationship across more than 100 phenotypes with greater power and fewer false positives [131]. Another such generalized suite of tools called Fusion has been developed and made publicly available by Gusev et al. (available at http://gusevlab.org/projects/fusion/).

The current TWAS approaches train separate imputation models for each tissue, which means information across tissues can be lost and the burden of multiple tests reduces statistical power to identify significant associations. A new method, UTMOST (unified test for molecular signatures), combines multiple single-tissue associations from SNP and GTEx eQTL data to increase accuracy and quantity of identified gene-trait associations [132]. This method can improve statistical power not only by reducing multiple-testing burden but also by increasing effective sample size since it considers similarities in transcriptional regulation across multiple tissues, some of which may be difficult to obtain for human populations. This method first imputes gene expression values per tissue via *cross-tissue* expression imputation (using penalized multivariate regression with group LASSO penalty), then tests for associations between trait and imputed expression in each tissue, and finally uses a statistical test to combine all the single-tissue gene-trait associations. A conceptually similar approach called MultiXcan was developed by Barbeira et al. [133] that uses multivariate regression to simultaneously regress the phenotype on predicted/imputed gene expression obtained *independently* from each of multiple tissues (using the standard elastic net penalty on each tissue). Most recently, the fine-mapping approach, FOCUS (fine-mapping of causal gene sets), was released as a way to perform statistical fine-mapping over gene-trait association signals from TWAS [134]. Finally, a useful perspective piece that discusses challenges and best practices for TWAS was recently published [135].

### Data integration strategies for annotating the non-coding regions of the genome

Many contemporary methods for annotating non-coding regions have employed data from multiple sources, including but not limited to ENCODE and GTEx. A number of tools have been developed to identify where SNPs reside with respect to non-coding features but one of the most popular is ANNOVAR, which is both fast, flexible, and can even annotate coding regions as well [52]. Two methods for specifically analyzing GWAS data that have successfully integrated many forms of functional genomics annotations include INFERNO and FUMA (Table 1). INFERNO (INFERring the molecular mechanisms of Noncoding genetic variants) is a method to annotate GWAS summary statistics by identifying nearby SNPs that are likely causal based on GTEx eQTLs, GENCODE annotations, FANTOM5, ENCODE, and NIH Roadmap data [136]. INFERNO successfully annotated GWAS variants associated with Schizophrenia or IBD in a tissue-specific manner. FUMA GWAS (Functional Mapping and annotation of Genome-Wide Association Studies) represents a tool that can annotate SNPs with genes or pathways using many of the same publicly available datasets as INFERNO [137]. Some difference between these methods include how eQTLs are utilized, and the use of functional annotations such as CADD score and ANNOVAR. Both methods have online interfaces that are easy to use and can utilize summary level data. As opposed to annotating the SNPs independently and within a single phenotype context, a group recently analyzed SNPs from the GWAS and PheWAS catalog utilizing data from the 1000 genomes project, FANTOM5, ENCODE, NIH Roadmap, and GTEx in a network based study [138].

While analysis of common variants that impact expression using TWAS and similar methods (Table 1) have identified many putative causal variants, there are still other forms of genetic variation that contribute to disease such as rare variants [139]. These rare SNPs, which are represented in less than 1% of the cohort or population of interest, are typically inherited independently of other nearby SNPs [140]. They also require different methods to analyze since they occur so infrequently in the same location across individuals, hence being “rare”. A paper from the GTEx consortium found that genes that are outliers with respect to expression (i.e. over- or under-expressed) are enriched with nearby rare variants when compared to non-outliers [141]. While it was mentioned previously that programs like VEP can be used to annotate coding features independently of expression, in this work, they also leveraged gene expression data to find which types of variants are enriched with these expression outliers. Finally, the group developed a Bayesian statistical model (RIVER) that incorporates expression data, along with annotations from VEP, Roadmap Epigenomics data, and CADD to predict the regulatory effect of the SNP [141]. Though the prediction accuracy was not extremely high (AUC = 0.64), it was significantly greater than using genomic information (AUC = 0.54) and highlights the utility of incorporating expression data when studying rare variants. While CADD scores can be very useful they are often now used in concert with other scoring methods when determining the functional impact of SNPs. It should be noted that LINSIGHT was able to outperform CADD when it came to characterizing non-coding regions associated with inherited diseases [142]. LINSIGHT uses a generalized linear model that primarily relies upon DNA conservation to make predictions, however the authors suggest the method could be adapted for deep learning.

### The future of non-coding annotations and data integration

While GTEx, ENCODE, and other resources have well characterized genetic variants, there are still many avenues of annotating non-coding regions that have not been thoroughly investigated. As previously mentioned, the biggest weakness of ENCODE was the lack of genetic variability between samples. And while the strength of GTEx was that genetic variance could be analyzed, it only generated resources for genetic and expression data. In order to develop a resource that has DNA accessibility, epigenetic marks, expression, post-transcriptional information, and proteomic data across multiple individuals and tissue types the GTEx consortium has proposed eGTEx (Enhancing GTEx), the next phase in generating data for GTEx [143]. In addition to SNPs that regulate protein coding genes, there has been a growing interest in annotating microRNAs (miRNA). Though methods like VEP can annotate miRNAs, there are more specialized tools that can incorporate RNA binding protein information, adjacent sequences, and external databases that contain information about the potential for a variant to perturb miRNA-mediated gene regulation [65]. Though these methods have proven useful on their own it will be interesting to see how they can be incorporated into high-dimensional data integration studies to analyze eQTLs. Finally, DNA can form conformations such as a non-canonical G-quadruplex, which have been illustrated to be highly influential in tuning gene expression [144]. While it has not been used to annotate SNPs across a wide-variety of traits, they have recently been implicated in helping explain the relationship between Alzheimer’s Disease and Herpesvirus [145]. It will be important to include G-quadruplex annotations in future analyses to characterize how generalizable its utility is for regulating gene expression and how often SNPs that affect its conformation impact a trait or disease.

## Emerging strategies for SNP annotations

### Single-cell RNA-sequencing

While bulk sequencing takes a sample or tissue and measures genetic, transcriptomic, and other forms of molecular variation on average across cell types, single-cell sequencing has enabled scientists to measure cell-type specific effects. Single-cell sequencing has been incredibly useful for those dissecting heterogeneity in cancer, as reviewed previously [146]. However, many challenges still exist for calling SNPs across the genome within single-cell data which have been reviewed elsewhere [147, 148]. Having said that, a number of recent publications have been able to succeed at using single-cell RNA-seq (scRNA-seq) data to analyze SNPs. scRNA-seq provided the resolution to suggest cis-acting variation may play a role in the level of expression on the inactivated X chromosome for an individual [149]. Allele specific expression in single-cell studies has been investigated in human fibroblasts [150] and dissect subclonal structure within chronic lymphocytic leukemia, but this used a more targeted approach [151]. Additionally, Poirion and colleagues were able to identify single-nucleotide variants along with measuring gene expression from scRNA-seq data for the purpose of characterizing cell subpopulations [152]. It is also possible to integrate GWAS data with single-cell studies to uncover novel relationships. For instance, mouse single-cell chromatin accessibility data has been used to investigate summary level GWAS data [153]. It will be exciting to see how single-cell methods are incorporated into currently available or new computational software in the future to analyze SNPs.

### Emerging strategies using deep learning

Deep learning is a sub-category within machine-learning that has recently begun to show promise in many biomedical fields, including that of annotating SNPs. For instance, Xiong et al. were able to use deep learning algorithms to generate scores that predict how much a variant will impact splicing [154]. To test the potential utility of their method for identifying SNVs that are pathogenic, they compared rare variants in highly expressed genes between controls and individuals with autism spectrum disorder (ASD). Interestingly, they were not able to find a difference among frequently implicated genes connected to ASD, but they did find significant differences between the genes predicted to have mis-spliced transcripts based on their method. Many of these novel hits had known connections to ontologies related to neurological phenotypes, suggesting they are robust candidate genes for future work. These results help illustrate that DL can be a useful framework for making sense of heterogenous relationships between SNPs and phenotypes which may have implications for translational research and precision medicine. Another avenue of research where DL has been making progress is annotating regulatory regions such as transcription-factor binding sites, enhancers, promoters, and microRNA binding sites in order to regulate gene expression [38]. Though these methods may be incorporating genetic information, they often are not designed to specifically annotate SNPs. On the other hand, DeepSEA is able to annotate regulatory elements by incorporating ENCODE data into a deep convolution network [155]. The method can then prioritize functionally relevant non-coding variants. The same group which developed DeepSEA has also developed ab initio, a tool which uses a DL framework to annotate the effects of mutations with tissue-specific gene expression context [156]. Recently, deep neural networks were used to improve clinical classification of disease causing variants by incorporating variation from other primate species into the model [157].

### High-throughput experimental assays can help inform SNP annotation

A rapidly evolving area of research that can be applied to SNP annotations are genetic screens. Genetic screens often test hundreds to thousands of genetic variants for some functional effect in a controlled manner [158]. This process is different than previous mentioned studies as instead of looking at all regions of the genome, or computationally predicting effects, many of these massively parallel reporter assays (MPRAs) test many of the same type of genetic element, such as variants that affect the promoters or enhancers of genes [159–161]. Likewise, they could test all of the coding variants in a gene of interest and using a reporter, display how a certain function of the gene is impacted by genetic variation [162]. While MPRAs are just beginning to gain traction in human genetics, they are becoming a powerful hypothesis driven and mechanistic approach to uncovering causal variants [163]. Thus, they will likely be more often incorporated into SNP annotation tools in the future. In fact, the Critical Assessment of Genome Interpretation (CAGI), a community platform for assessing computational methods that predict phenotypes from genomic variant data, already includes challenges that use these high-throughput screens to evaluate algorithms (https://genomeinterpretation.org/content/5-challenge).

## Conclusions

In summary, our understanding of how genetic variation influences traits and diseases has rapidly evolved in recent years. A number of large studies that have pooled resources together have generated sequencing and phenotypic data. These studies have highlighted how we can traverse between characterizing how SNPs and phenotypes are related. SNP classification systems often differ depending on whether SNPs are non-synonymous, synonymous, or non-coding. Additionally, interpretation of the annotation results can vary not only because of the methodology, but also based on whether the results are used for research or clinical purposes. One of the biggest obstacles for the field for those doing more basic research is to find the sources of omics data that are most important and incorporating these into new methods, while those performing more applied or clinical studies need to more accurately identify SNPs most closely associated with disease in order to move them into clinical or pharmacogenomic use. In order to accomplish this goal, it will be important for researchers to incorporate more ethnically diverse annotations so that potential therapies are not limited to individuals of European ancestry [164]. Finally, trait and disease risk SNPs are becoming a topic of discussion in the public; thus, it is important that scientists are able to effectively communicate the implications and limitations of their efforts to the public. It is important to arm the public with this knowledge so they can decide if they should incorporate genetic testing as a means to achieve improved health outcomes.
